# Hsa_circ_0007967 promotes gastric cancer proliferation through the miR-411-5p/MAML3 axis

**DOI:** 10.1038/s41420-022-00954-1

**Published:** 2022-03-30

**Authors:** Quanbin Zha, Xi Wu, Jingxin Zhang, Tingting Xu, YongKang Shi, Yayun Sun, Yuan Fang, Yunru Gu, Pei Ma, Yongqian Shu, Shengwang Tian

**Affiliations:** 1 Department of Oncology, Jintan Hospital, Jiangsu University, Changzhou, 213200 People’s Republic of China; 2 Department of Oncology, the First Affifiliated Hospital of Nanjing Medical University, Nanjing, 210029 People’s Republic of China; 3 Department of General Surgery, the Affifiliated People’s Hospital of Jiangsu University, Zhenjiang Clinic School of Nanjing Medical University, Zhenjiang, 212002 People’s Republic of China; 4 Department of Neurology, Jintan Hospital, Jiangsu University, Changzhou, 213200 People’s Republic of China; 5 Department of Oncology, Sir Run Run Hospital, Nanjing Medical University, Nanjing, 210029 People’s Republic of China; 6 Jiangsu Key Lab of Cancer Biomarkers, Prevention and Treatment, Collaborative Innovation Center for Cancer Personalized Medicine, Nanjing Medical University, Nanjing, 210029 People’s Republic of China

**Keywords:** Gastric cancer, Gastric cancer

## Abstract

Circular RNAs are an important kind of noncoding RNAs and involved in cancerogenesis, but the specific mechanism between gastric cancer and circRNAs needs further study. Hsa_circ_0007967 was selected by RNA sequencing. Here, hsa_circ_0007967 was highly expressed in gastric cancer tissues than adjacent normal tissues. Overexpressing hsa_circ_0007967 promoted gastric cancer cell proliferation in vitro and in vivo, while suppression of hsa_circ_0007967 inhibited gastric cancer cell proliferation in vitro and in vivo. Mechanistically, hsa_circ_0007967 sponged miR-411-5p to increase MAML3 expression. Overall, hsa_circ_0007967 is a promising biomarker for gastric cancer diagnosis and a potential molecule for gastric cancer treatment.

## Introduction

Gastric cancer (GC) is a significant public health problem because of its high morbidity and mortality. Across the world, the prevalence is much higher in Asia, Africa, South America, and Eastern Europe [1]. With the development of advanced technology, targeted drugs, and immune checkpoint inhibitors are emerging, but their effect on advanced GC patients is unsatisfactory, and the 5-year survival rate of such patients is <10% [2], which is mainly caused by the recurrence and metastasis. It is important for patients to accept early diagnosis and prevent metastasis. However, the specificity and sensibility of clinical traditional test like serum tumor markers is low [3], so it is meaningful to further investigate mechanisms behind GC progression and provide new sights for diagnosis and treatment.

As an important kind of non-coding RNAs, circular RNAs (cirRNAs) exhibit closed circular structures without free 3′ and 5′ tails, which are resistant to nuclease and make circRNAs stable [4]. Owing to the development of high-throughput sequencing and bio-informatics, more knowledge about circRNAs is being excavated. CirRNAs can not only function as miRNA sponges [5], but also act as transcription and translation regulaters [6–9], as well as become scaffolds to facilitate the interaction between protein [10–13]. What’s more, specific circRNAs could encoding protein [14–16]. Emerging studies suggest that the aberrant expression of cirRNAs may lead to a variety of diseases, such as cancers, cardiovascular system diseases, and nervous system diseases [17].

In our study, we performed RNA-seq between GC tissues and matched normal tissues and identified hsa_circ_0007967. We demonstrated that hsa_circ_0007967 promoted GC proliferation through miR-411-5p/MAML3 axis in vitro and in vivo.

## Results

### Identification and characterization of hsa_circ_0007967

**Fig. 1:**
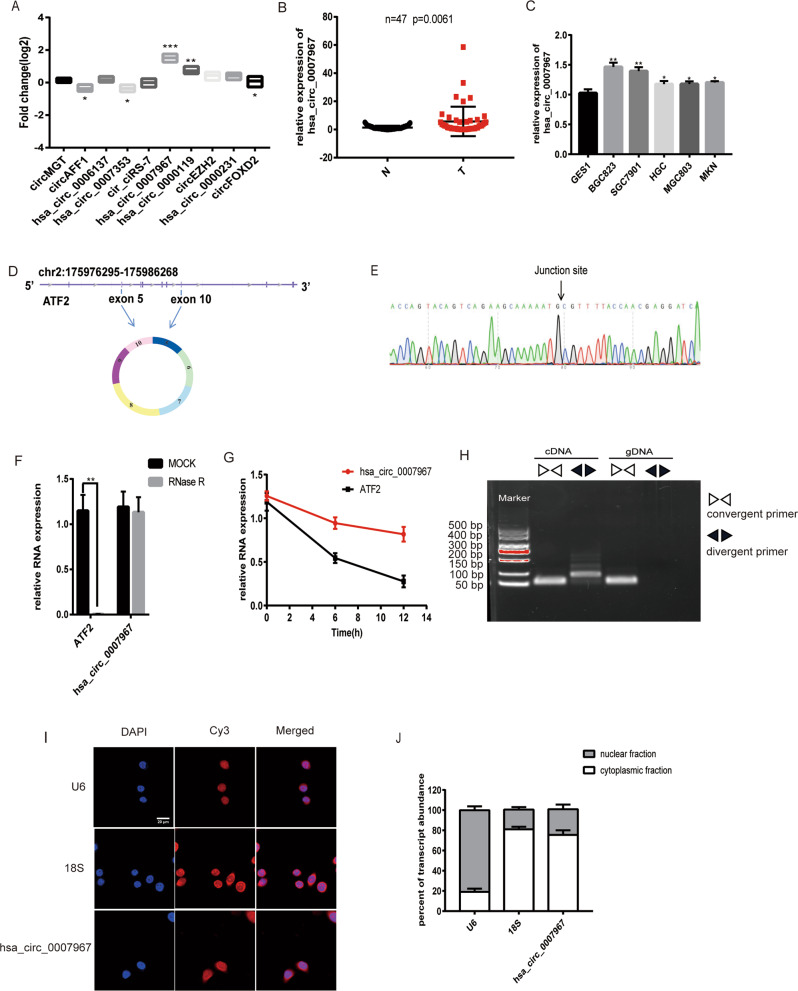
Identification and characterization of hsa_circ_0007967. **A** Expression of five most upregulated and downregulated circRNAs in five paired GC tumors and normal tissues. **B** Expression of hsa_circ_0007967 in 47 pairs of GC tissues and normal tissues. **C** Expression of hsa_circ_0007967 in GC cell lines and normal gastric epithelial cell line. **D** The location of hsa_circ_0007967 in chromatin and its structure. **E** Sanger sequencing confirming the back splicing junction site. **F** The RNase R digestion assay suggesting hsa_circ_0007967 is resistant to RNase R. **G** The use of act-D (1 μg/μl) demonstrating that hsa_circ_0007967 is stable than its linear form. **H** Northern blotting of hsa_circ_0007967 and its linear form in cDNA and gDNA amplified by convergent and divergent primers. **I** Location of hsa_circ_0007967 in BGC823 cells detected by FISH. **J** QPCR analysis of nuclear and cytoplasmic fractions conforming that hsa_circ_0007967 is located in cytoplasm.

To study how circRNAs involved in GC progression, 5 pairs of GC tissues and normal tissues were collected for RNA sequencing. A total of 20 circRNAs were differently expressed in GC tissues (fold change > 5), among which 10 were upregulated and 10 were downregulated. We evaluated the expression of top 5 upregulated and downregulated cirRNAs in forementioned 5 pairs of tissues. It was found that only hsa_circ_0007967 exhibited the most significant change (Fig. 1A). Then we evaluated the expression of hsa_circ_0007967 in another 47 pairs of GC tissues and matched normal tissues by RT-qPCR, and the expression of hsa_circ_0007967 was significantly higher in GC tissues (Fig. 1B). Consistently, hsa_circ_0007967 was higher expressed in GC cell lines (SGC7901, MGC803, BGC823, HGC27, AGS, and MKN87) than in the normal gastric epithelial cell line GES1 (Fig. 1C).

Hsa_circ_0007967 (chr2:175976295-175986268) was back spliced from 5 to 10 exons of protein-coding gene ATF2 (Fig. 1D). Its circular structure was confirmed by sanger sequencing (Fig. 1E). As well, RNase R digestion assays and the use of act-D (1 μg/μl) suggested that hsa_circ_0007967 was more stable and had a lower degradation rate compared with the corresponding mRNA (Fig. 1F, G). Additionally, hsa_circ_0007967 could be amplified from cDNA and gDNA by convergent primers, but only cDNA by divergent primers (Fig. 1H). Finally, fluorescence in situ hybridization (FISH) and RT-qPCR assays of nuclear and cytoplasmic fractions confirmed that hsa_circ_0007967 mainly located in cytoplasm (Fig. 1I, J). These results suggested that hsa_circ_0007967 was upregulated in GC tissues, and was a stable circRNA in cytoplasm.

### Hsa_circ_0007967 promotes GC cell proliferation in vitro and in vivo

**Fig. 2:**
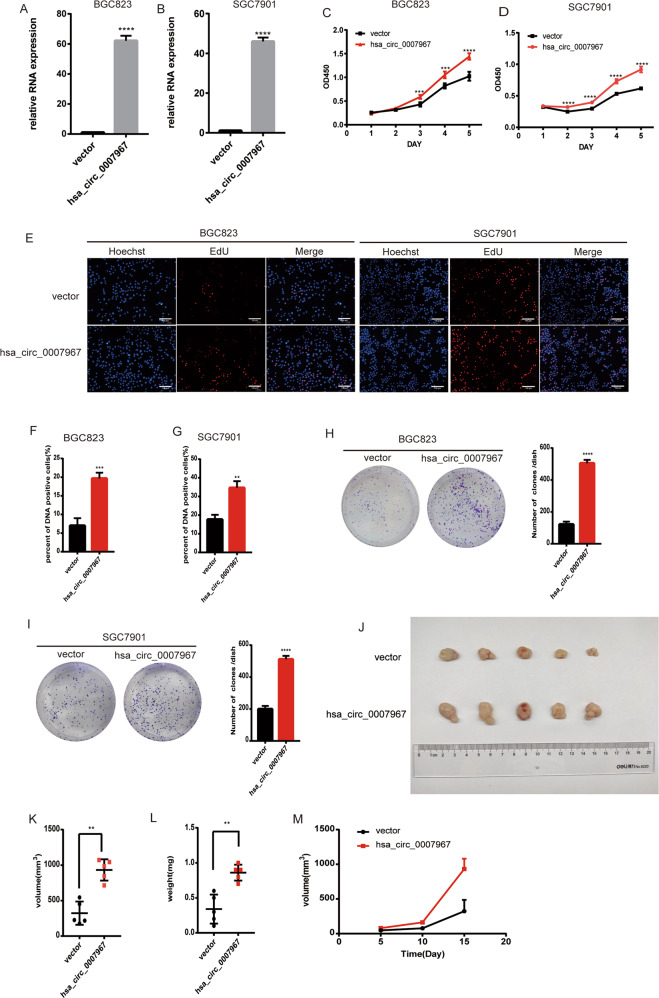
Overexpressing hsa_circ_0007967 promotes GC proliferation in vitro and in vivo. **A**, **B** The expression of hsa_circ_0007967 in BGC823 and SGC7901 cells transfected with vector and hsa_circ_0007967 plasmids. **C**, **D** The growth rate of BGC823 and SGC7901 cells transfected with vector and hsa_circ-0007967 by cck8 assays. **E** EdU assays of BGC823 and SGC7901 cells transfected with vector and hsa_circ-0007967 plasmids. **F**, **G** DNA positive cells of BGC823 and SGC7901 cells transfected with vector and hsa_circ-0007967 by EdU assays. **H**, **I** The assessment of proliferation of BGC823 and SGC7901 cells transfected with vector and hsa_circ_0007967 plasmid by colony formation assays. **J**–**L** Tumors from mice in two different groups, and the weight and volume of tumors at the end point. **M** The weight of tumors measured every 5 days until the end point.

**Fig. 3:**
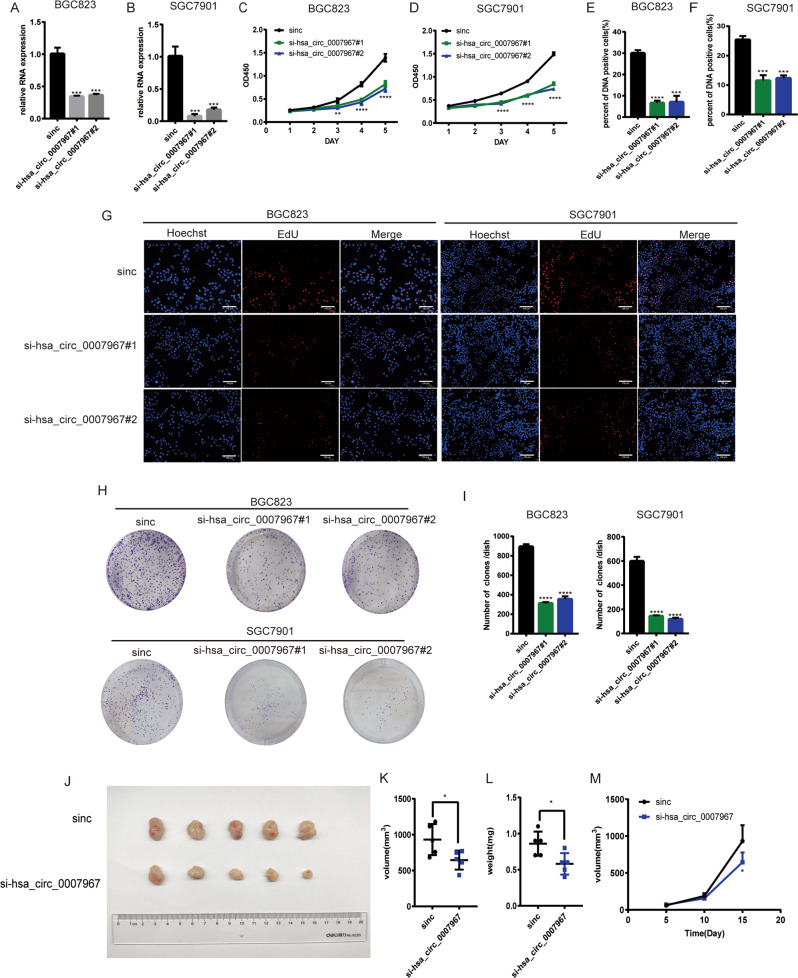
Silencing hsa_circ_0007967 inhibits GC proliferation in vitro and in vivo. **A**, **B** The expression of hsa_circ_0007967 in BGC823 and SGC7901 cells transfected with sinc, si-hsa_circ_0007967#1, and si-hsa_circ_0007967#2. **C**, **D** The growth rate of BGC823 and SGC7901 cells transfected with sinc, si-hsa_circ-0007967#1, and si-hsa_circ-0007967#2 by cck8 assays. **E**, **F** DNA positive cells of BGC823 and SGC7901 cells transfected with sinc, si-hsa_circ-0007967#1, and si-hsa_circ-0007967#2 by EdU assays. **G** EdU assays of BGC823 and SGC7901 cells transfected with sinc, si-hsa_circ-0007967#1, and si-hsa_circ-0007967#2. **H**, **I** The assessment of proliferation of BGC823 and SGC7901 cells transfected with sinc, si-hsa_circ_0007967#1, and si-hsa_circ_0007967#2 by colony formation assays. **J**–**L** Tumors from mice in two different groups, and the weight and volume of tumors at the end point. **M** The weight of tumors measured every 5 days until the end point.

To explore whether hsa_circ_0007967 involves in the progression of GC, we first overexpressed hsa_circ_0007967 in BGC823 and SGC7901 cells (Fig. 2A, B). Cck8 assays showed that cells with hsa_circ_0007967 overexpressed had a faster growth rate (Fig. 2C, D). 5-Ethynyl-2′-deoxyuridine (EdU) assays suggested that the percent of DNA positive cells was higher in hsa_circ_0007967-overexpressed cells (Fig. 2E–G). Consistently, colony formation assays showed that hsa_circ_0007967-overexpressed cells grew into bigger clones (Fig. 2H, I). Then, we downregulated the expression of hsa_circ_0007967 in BGC823 and SGC7901 with 2 siRNAs (Fig. 3A, B). On the contrary, Cck8, EdU, and colony formation assays revealed that silencing hsa_circ_0007967 could inhibit GC cell proliferation (Fig. 3C–I). To further investigate whether hsa_circ_0007967 could accelerate the proliferation of GC cell in vivo, twenty BALB/c-nude mice were divided randomly into 4 groups and mice in different groups were subcutaneously injected with differently treated BGC823 cells (hsa_circ_0007967-overexpressed, hsa_circ_0007967-silenced, and the correspond control ones). Mice that were injected with hsa_circ_0007967-overexpressed BGC823 cells bore bigger and heavier tumors than those in the control group (Fig. 2J–M). And the weight and volume of tumors in the mice that were injected with hsa_circ_0007967-downregulated BGC823 cells were significantly lighter and smaller than those in the control group (Fig. 3J–M). These data suggested that hsa_circ_0007967 facilitated GC cells proliferation in vitro *and* in vivo.

### Hsa_circ_0007967 serves as the sponge of miR-411-5p

**Fig. 4:**
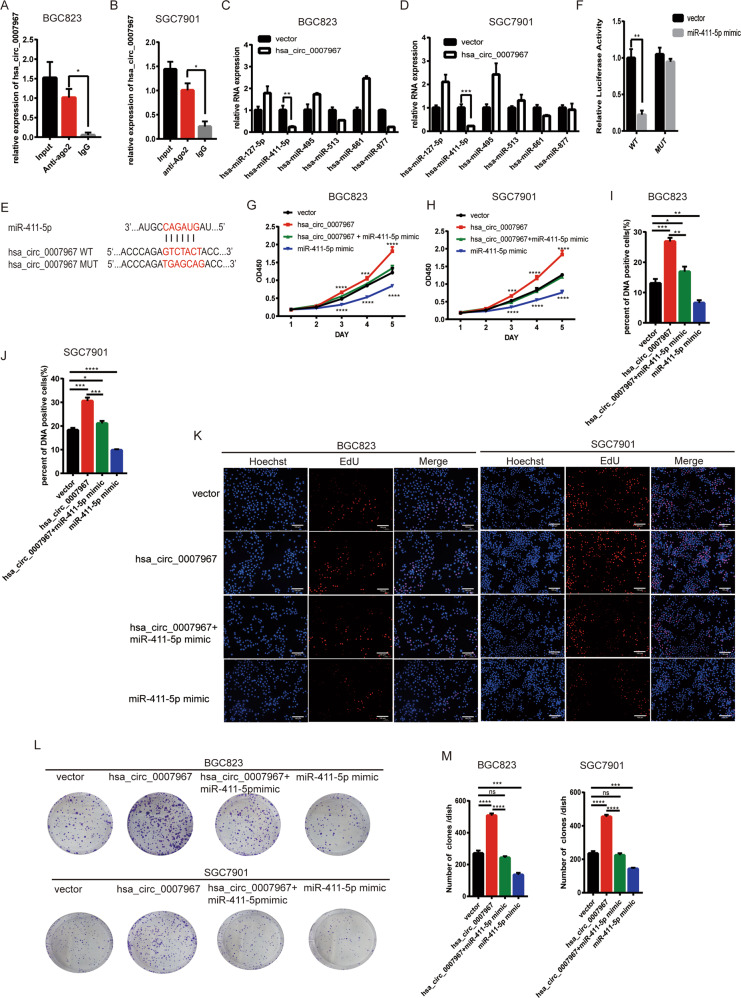
Hsa_circ_0007967 serves as the sponge of miR-411-5p. **A**, **B** Level of hsa_circ_0007967 enriched for Ago2 by RIP assays in BGC823 and SGC7901 cells. **C**, **D** The expression of selected miRNAs in BGC823 and SGC7901 cells after overexpressing hsa_circ_0007967. **E** The potential binding site between miR-411-5p and hsa_circ_0007967. **F** The relative luciferase activity in HEK-293T cells cotransfected with miR-411-5p mimics and hsa_circ_0007967 plasmids with WT/MUT binding site by Dual luciferase reporter assays. **G**, **H** The growth rate of BGC823 and SGC7901 cells transfected with vector, hsa_circ_0007967 plasmids, miR-411-5p mimics or cotransfected with hsa_circ_0007967 plasmids and miR-411-5p mimics by cck8 assays. **I**–**K** DNA positive cells of BGC823 and SGC7901 cells transfected with vector, hsa_circ_0007967 plasmids, miR-411-5p mimics or cotransfected with hsa_circ_0007967 plasmids and miR-411-5p mimics by EdU assays. **L**, **M** The assessment of proliferation of BGC823 and SGC7901 cells transfected with vector and hsa_circ_0007967 plasmids by colony formation assays.

As hsa_circ_0007967 is mainly located in the cytoplasm, we speculated that hsa_circ_0007967 may act as miRNA sponges. We performed RNA-protein immunoprecipitation (RIP) assays and found that more hsa_circ_0007967 was enriched with anti-AGO2 antibody than IgG, which suggested that hsa_circ_0007967 bound well to miRNAs (Fig. 4A, B). Then we searched CircInteractome (circinteractome.nia.nih.gov) and chose six potential miRNAs with highest score (miR-127-5p, miR-411-5p, miR-495, miR-611, miR-513, and miR-877). Overexpressing hsa_circ_0007967 only caused a significant downregulation of miR-411-5p in both BGC823 and SGC7901 cells, suggesting that miR-411-5p may be the downstream molecule of hsa_circ_0007967 (Fig. 4C, D). We predicted the binding site between hsa_circ_0007967 and miR-411-5p (Fig. 4E). Dual luciferase reporter assays showed that miR-411-5p mimics only caused a significant reduction of the relative luciferase activity in hsa_circ_0007967-WT group (Fig. 4F). To further study whether hsa_circ_0007967 promoted GC proliferation by regulating miR-411-5p, we performed rescue experiments. Cck8, EdU, and colony formulation assays showed that miR-411-5p mimics inhibited GC cell proliferation, and suppressed GC cell proliferation caused by hsa_circ_0007967 overexpression (Fig. 4G–M).

### MAML3 is the target gene of miR-411-5p and promotes GC progression

**Fig. 5:**
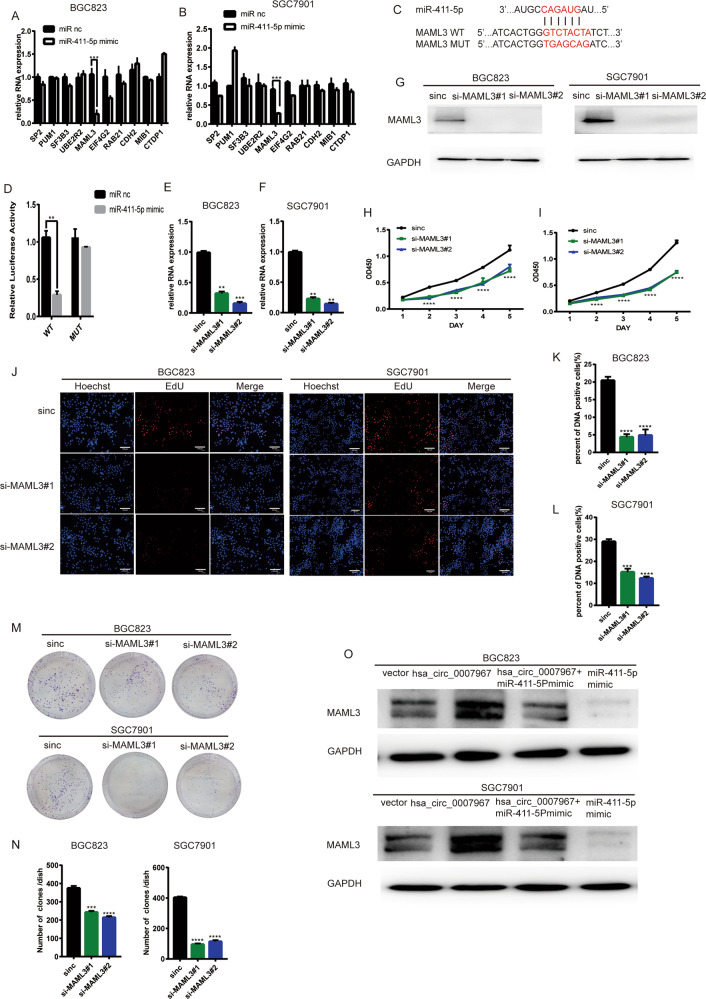
MAML3 is the target gene of miR-411-5p and promotes GC progression. **A**, **B** level of selected genes in BGC823 and SGC7901 cells transfected with miR-411-5p mimics. **C** The potential binding site between miR-411-5p and MAML3 mRNA. **D** The relative luciferase activity in HEK-293T cells cotransfected with miR-411-5p mimics and MAML3 plasmids with WT/MUT binding site by Dual luciferase reporter assays. **E**, **F** The expression of MAML3 in BGC823 and SGC7901 cells transfected with sinc, si-MAML3#1 and si-MAML3#2 by qPCR. **G** The expression of MAML3 in BGC823 and SGC7901 cells transfected with sinc, si-MAML3#1 and si-MAML3#2 by WB. **H**, **I** The growth rate of BGC823 and SGC7901 cells transfected with sinc, si-MAML3#1, siMAML3#2 by cck8 assays. **J**–**L** DNA positive cells of BGC823 and SGC7901 cells transfected with sinc, si-MAML3#1, siMAML3#2 by EdU assays. **M**, **N** The assessment of proliferation of BGC823 and SGC7901 cells transfected with sinc, si-MAML3#1 and si-MAML3#2 by colony formation assays. **O** The protein level of MAML3 of BGC823 and SGC7901 cells transfected with vector, hsa_circ_0007967 plasmids, miR-411-5p mimics or cotransfected with hsa_circ_0007967 plasmids and miR-411-5p mimics by WB.

MiRNAs generally bind to the 3′ untranslated region of mRNAs and lead to mRNA degradation [18]. After searching three websites (TargetScan, TargetMiner, and miRDB), we found 12 mutual protein candidates that miR-411-5p targets. While miR-411-5p mimics only caused MAML3 downregulate in both BGC823 and SGC7901 cells (Fig. 5A, B). MAML3 is encoded by Mastermind like (MAML) family genes and is an important transcriptional co-activator in the Notch signaling pathway. The aberrant expression of MAML3 may lead to different diseases, like cancers [19]. Then, we conducted dual luciferase reporter assays with mutant and wild-type MAML3 plasmids (Fig. 5C). Dual Luciferase reporter assays showed that miR-411-5p mimics reduced the activity of luciferase in the WT group, which suggested that miR-411-5p bound to the 3’UTR of MAML3 mRNA (Fig. 5D). To further investigate whether MAML3 was involved in GC proliferation, we silenced MAML3 in both BCG823 and SGC7901 cells, which was confirmed in mRNA and protein level (Fig. 5E–G). Cck8, EdU, and colony formation assays suggested that silencing MAML3 inhibited GC proliferation (Fig. 5H–N). What’s more, WB assays suggested that overexpressing hsa_circ_0007967 could cause the high expression of MAML3 and miR-411-5p mimics downregulated the expression of MAML3. While miR-411-5p mimics suppressed the upregulation of MAML3 caused by hsa_circ_0007967 overexpression (Fig. 5O). Overall, hsa_circ_0007967 promoted GC proliferation through miR-411-5p/MAML3 axis.

## Discussion

In recent years, emerging studies suggested that circRNAs play an important role in cancerogenesis. Here, we identified the oncogenic circRNA, hsa_circ_0007967, through RNA-seq, which was highly expressed in GC tissues than in matched normal tissues. It was back spliced from ATF2 gene and mainly located in cytoplasm. Cck8, EdU, and colony formation assays demonstrated that overexpressing hsa_circ_0007967 promoted GC proliferation in vitro. Consistently, overexpressing hsa_circ_0007967 promoted subcutaneously injected GC cells proliferation. Hsa_circ_0007967 was the sponge of miR-411-5p and finally upregulated MAML3 expression. Overexpressing miR-411-5p suppressed GC proliferation and weakened the oncogenic effect of hsa_circ_0007967. MAML3 is an important transcriptional co-activator in the Notch signaling pathway and silencing MAML3 suppressed GC proliferation. Overexpressing hsa_circ_0007967 caused the upregulation of MAML3, which was resecured by overexpressing miR-411-5p. It was suggested that hsa_circ_0007967/miR-411-5p/MAML3 axis involved in GC progression. Consequently, hsa_circ_0007967 is a promising biomarker for GC diagnosis and prognosis, and is a potential therapeutic target for GC treatment.

Though our study demonstrated that hsa_circ_0007967 played an oncogenic role in GC, we only investigated that hsa_circ_0007967 functioned as an miRNA sponge and just focused on the malignant activity of proliferation without further exploration. What’s more, we only evaluated the expression of circRNAs between tumors and adjacent non-malignant tissues. It would be better to conduct an appropriate control cohort that reflected the intended use of the biomarker. We believed that, with the development of technology, circRNA expression will oneday be detected at the single-cell level and with spatial resolution, which will be essential for better understanding circRNA functions in the future.

## Materials and methods

### Patient samples

A total of 47 pairs of GC tissues and adjacent normal tissues were collected from the hospital of Zhenjiang according to institutional protocols and this study was approved by the Medical Ethics Committee of First Affiliated Hospital of Nanjing Medical University. Informed consent form was signed by every patient.

### Cell culture

The HEK-293T, MGC803, BGC823, HGC27, SGC7901, AGS, and GES1 cell lines were purchased from Type Culture Collection of the Chinese Academy of Sciences (Shanghai, China). The HEK-293T, MGC803, HGC27, BGC823, SGC7901, and GES1 cells were cultured in RPMI 1640 medium (Gibco, USA). The AGS cells were cultured in F12k medium (Wisent, Canada). All the cell lines were cultured in a 37 °C, 5% CO_2_ incubator (Thermo Fisher, USA), and were provided with 100 μg/ml streptomycin (Gibco), 100 U/ml penicillin (Gibco), and 10% fetal bovine serum (BI, Iseral).

### RNA extraction and quantitative real-time polymerase chain reaction

Total RNA was extracted from the cells or tissues using TRIzol reagent (Ambion, USA). The nuclear and cytoplasmic RNAs were extracted with PARIS™ Kit (Thermo Fisher, USA). Isolated RNAs were reversely transcribed into cDNAs with HiScript Q RT SuperMix for qPCR (Vazyme, China). RT-qPCR assays were carried out with SYBR Green PCR Master Mix (Vazyme, China) on the Applied Biosystems steponeplus (USA) Real Time PCR system. GAPDH and U6 were used as internal controls, and expressions of all samples were normalized to GAPDH and U6. The primers are shown in Table 1.

**Table 1:** Primers used in this study.

Names	Sequences (5′-3′)
Hsa_circ_0007967: forward	CCCTGTACCAGGCCCATTTC
Hsa_circ_0007967: reverse	TGGGACTGCAGCTGGAACA
MAML3: forward	CCTACCAGCCAACCAGGAATGTA
MAML3: reverse	ATGCTCTGACCAAAGCCACTCAC
miR-411-5p: forward	GGCCGGCTAGTAGACCGTATAG
miR-411-5p: reverse	ACTGCAGGGTCCGAGGTATT
GAPDH: forward	GGGAGCCAAAAGGGTCAT
GAPDH: reverse	GAGTCCTTCCACGATACCAA
U6: forward	CTCGCTTCGGCAGCACA
U6: reverse	AACGCTTCACGAATTTGCGT

### SiRNA and plasmid transfection

The linear form of hsa_circ_0007967 was inserted into plasmid pcDNA3.1-CMV by Hanbio Biotechnology (Shanghai, China). SiRNAs targeting hsa_circ_0007967 were purchased from RiboBio (Guangzhou, China). SiRNAs targeting MAML3 were purchased from GenePharma (Shanghai, China). The miRNA mimics or inhibitors were purchased from GenePharma. The plasmids, siRNAs, miRNA mimics, and inhibitors were transfected into cells with Lipofectamine 3000 (Life Technologies, USA).

### RNase R treatment

In all, 5 μg of total RNA was incubated for 15 min at 37 °C with or without 4 U/μg of RNase R (Epicentre Technologies, USA) in 1× reaction buffer, and was then reversely transcribed into cDNA.

### RNA fluorescence in situ hybridization

FISH assays were conducted with Ribo^TM^ Fluorescence In Situ Hybridization Kit (RiboBio) under the manufacturer’s instruction. Cy3-labeled probes targeting hsa_circ_0007967, U6, 18S were purchased from RiboBio. Cells were seeded into eight-well plate and incubated for 12 h before fixation. After 30 min’ fixation, and 10 min’ permeabilization (0.5% Triton X-100), cells were prehybridized in prehybridization buffer at 37 °C for half an hour. Then cells were hybridized in hybridization buffer with specific probes at 37 °C overnight in the dark. 4×SSC (including 0.1% Tween-20), 2×SSC and 1×SSC were used for washing off hybridization buffer at 42 °C in the dark. Confocal images were captured by Zeiss LSM5 confocal microscope (Carl Zeiss Jena, Oberkochen, Germany).

### Cell counting kit-8 assay

After 48 h of transfection, cells (2 × 10^3^/well) were seeded into 96-well plates (Corning,USA). Then 100 μl of 10% Cell counting kit-8 (CCK8; Beyotime, China) solution was added to each well at appointed time (8 h, 24 h, 48 h, 72 h, 96 h). After 2 h of incubation at 37 °C, the absorbance at 450 nm was measured with a microplate reader (Pro-11 multiskan FC, Thermo Fisher, USA).

### Colony formation assay

After 48 h of transfection, cells (1 × 10^3^/well) were seeded into six-well plates (Corning). After incubation for 10 days at 37 °C, cells were fixed with methyl alcohol and stained with crystal violet solution.

### 5**-**Ethynyl-2′-deoxyuridine incorporation assay

EdU assays were performed with the Cell-Light EdU DNA Cell Proliferation Kit (RiboBio) according to the manufacturer’s instruction. Images were obtained with a Nikon Ti microscope (Nikon, Tokyo, Japan), and the number of EdU positive cells was counted.

### Western blotting

Cells were lysed in RIPA buffer (Beyotime, China) with 1% PMSF (Biosharp, China). The protein was separated by sodium dodecyl sulfate-polyacrylamide gel electrophoresis (Epizyme, China) and transferred onto PVDF membrane (Millipore, USA). Primary antibodies were applied at 4 °C overnight and HRP-conjugated secondary antibodies were applied for an hour at room temperature. The immunocomplexes were detected with ECL Western Blotting Substrate (NCM Biotech, China), visualized with Tanon (5200multi 4600SF, Tanon, USA). GAPDH was used as the internal control. Primary Antibodies included rabbit antiMAML3 (1:500, Biorbyt, UK), mouse antiGAPDH (1:20000, Beyotime, China). Secondary antibodies (A0208 and A0216, Beyotime, China) were diluted in 1:1000.

### Northern blotting

DNA was separated using 1% agarose gel electrophoresis for 20 min under 110 v and was detected by BIO-RAD (BIO-RAD Gel Doc XR+, USA)

### RNA stability assay

Cells were seeded into 6-well plates for 12 h incubation and grew to 50% confluence. Then cells were treated with 1 μg/ml actinomycin D and total RNAs were collected at 0, 6, 12 h. RNA levels were detected using RT-qPCR, and the halflife of cirRNAs and mRNAs was evaluated.

### Dual luciferase reporter assay

The wild-type sequence of hsa_circ_0007967 and the 3′UTR of MAML3 which containing predicted binding site of miR-411-5p were subcloned into the luciferase reporter vector GV272 (GenePharma, China). The corresponding ones containing mutant predicted binding site of miR-411-5p were subcloned into the luciferase reporter vector GV272 (GenePharma). HEK-293T cells were seeded in 24-well plate (6 × 10^4^ cells/well) for 24 h before transfection. Cells were co-transfected with a mixture of luciferase reporter vectors containing wild-type sequence or mutant sequence along with miRNA mimics. After 24 h incubation, the luciferase activity was measured with a specific microplate reader (Synergy H1, USA). Dual-Luciferase®Reporter (DLR™) Assay System was used according to the manufacturer’s instructions.

### RNA-protein immunoprecipitation

The MagnaRIP RNA-Binding Protein Immunoprecipitation Kit (Merk, USA) was employed according to the manufacturer’s instructions. The cell lysate was incubated with beads coated with 5 μg of antibody against Argonaute-2 (AGO2) (Abcam, USA), and control IgG with rotation at 4 °C overnight. Total RNA was extracted for the evaluation of circRNA expression by RT-qPCR.

### Animal studies

All animal experiments were approved by the Institutional Animal Care and Use Committee of Nanjing Medical University. Twenty BALB/c-nude mice (female, 4-week-old) were divided randomly into 4 groups with online tool (Each was given a random number) and mice in different groups were injected with differently treated BGC823 cells (hsa_circ_0007967-overexpressed, hsa_circ_0007967-silenced and the correspond control ones). Cells (5 × 10^6^) were injected into the left back subcutaneously. The body weight and tumor volume (volume = length × width^2^/2) were measured every 5 days after injection until mice were killed. At the end of experiments, the mice were killed, and the tumors were dissected and weighed.

### Statistical analysis

GraphPad Prism software was used for statistical analysis. The data were presented as the mean ± standard deviation. Student’s *t* test was used for the determination of the statistical signifcance. A *p* value that <0.05 was considered statistically significant. Each experiment was repeated for three times with similar results.

## Supplementary information

Original Data File

## Data Availability

The data supporting the conclusion of this article are presented within the article and its additional files.
