# Supplementary material for: Hsa_circ_0007967 promotes gastric cancer proliferation through the miR-411-5p/MAML3 axis
Source: Cell Death Discov. 2022 Mar 30;8:144. doi: 10.1038/s41420-022-00954-1 (PMC8969178; doi:10.1038/s41420-022-00954-1)
Supplement: Supplementary file 1 — Original Data File [file 41420_2022_954_MOESM1_ESM.doc]

Figure 5 G


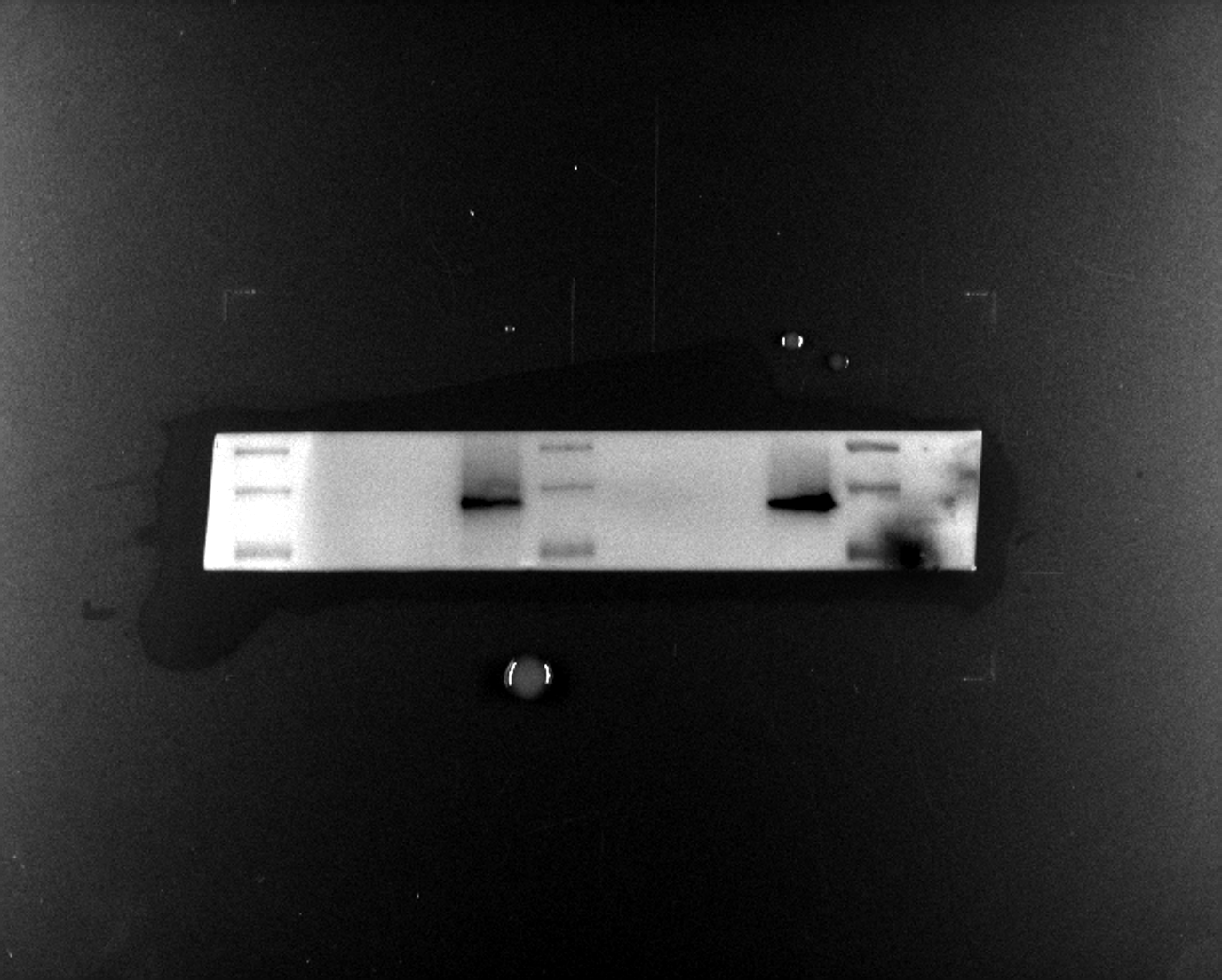

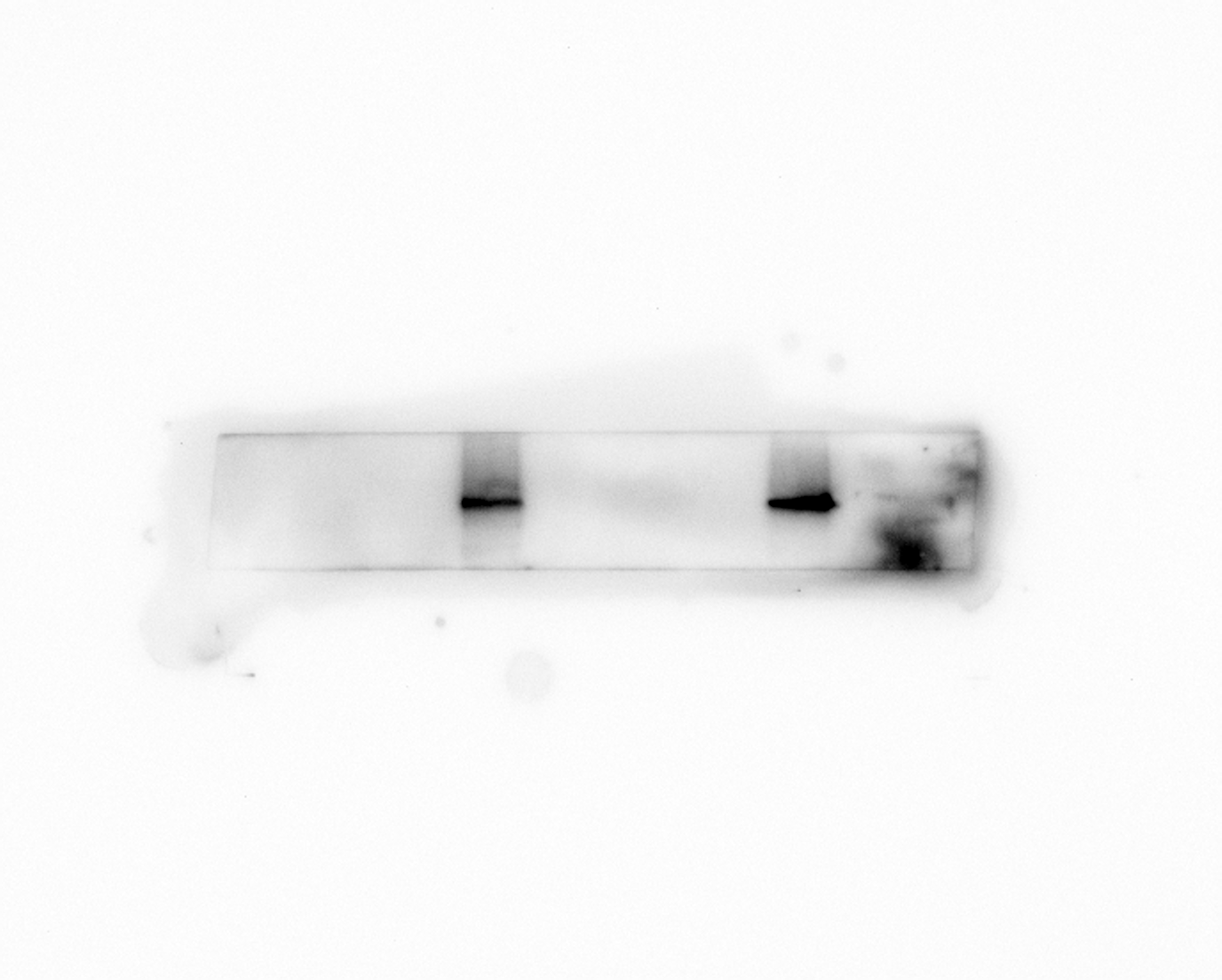


Antibody: MAML3

From right to left: MARKER, SGC7901nc, SGC7901si-MAML3#1, SGC7901si-MAML3#2, MARKER, BGC823nc,BGC823si-MAML3#1,BGC823si-MAML3#2


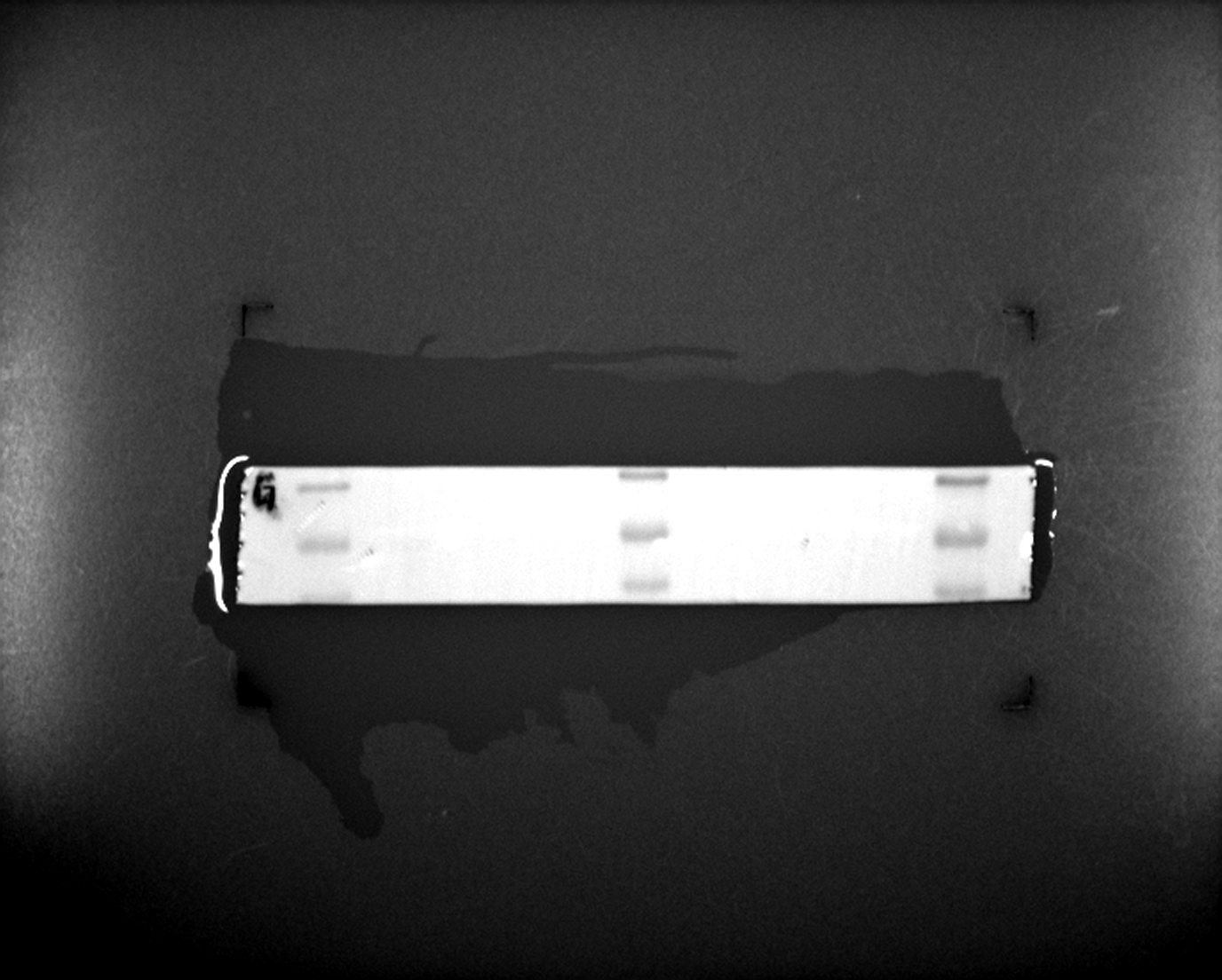

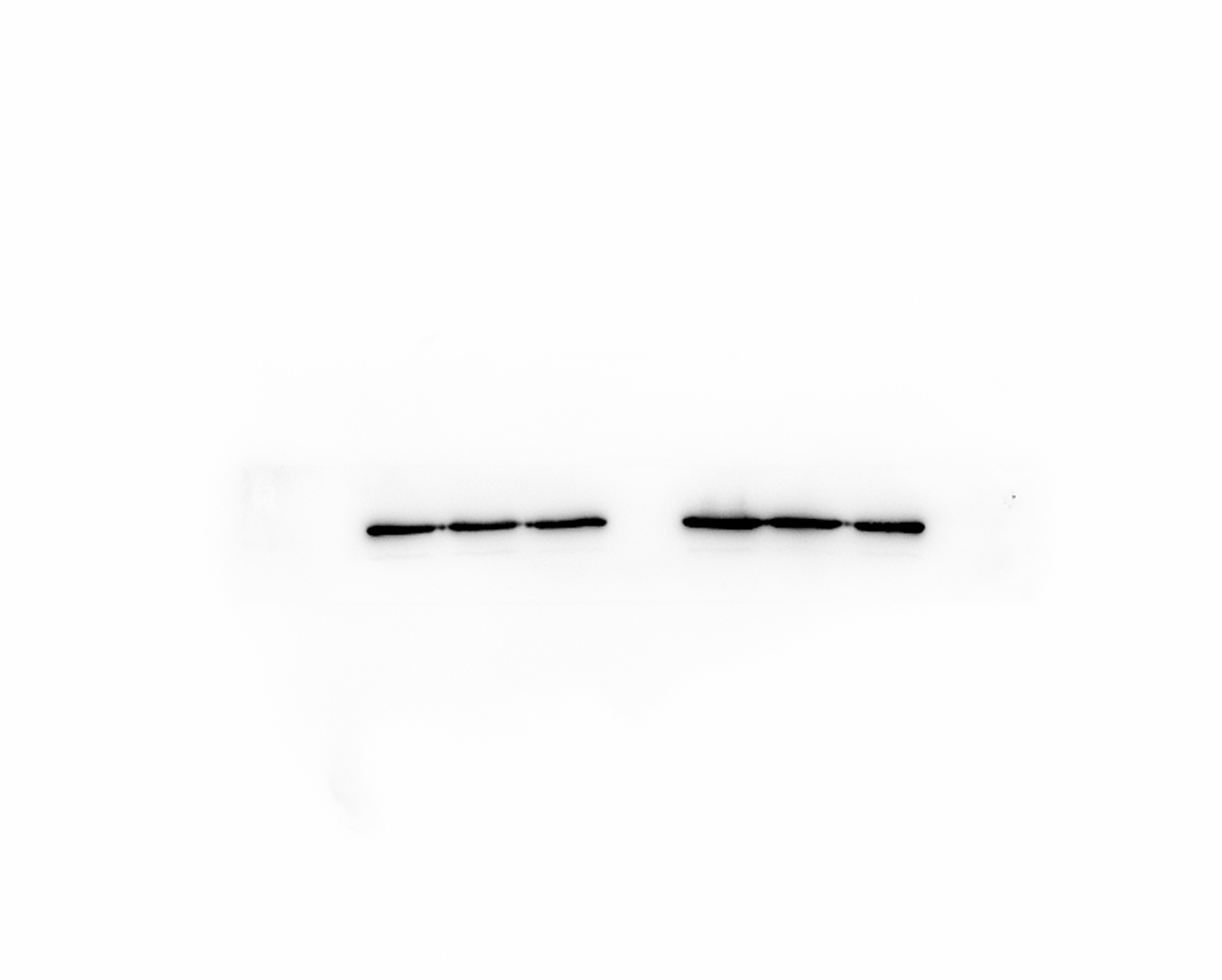


Antibody:GAPDH

From right to left: MARKER, SGC7901nc, SGC7901si-MAML3#1, SGC7901si-MAML3#2, MARKER, BGC823nc,BGC823si-MAML3#1,BGC823si-MAML3#2

Figure 5 O


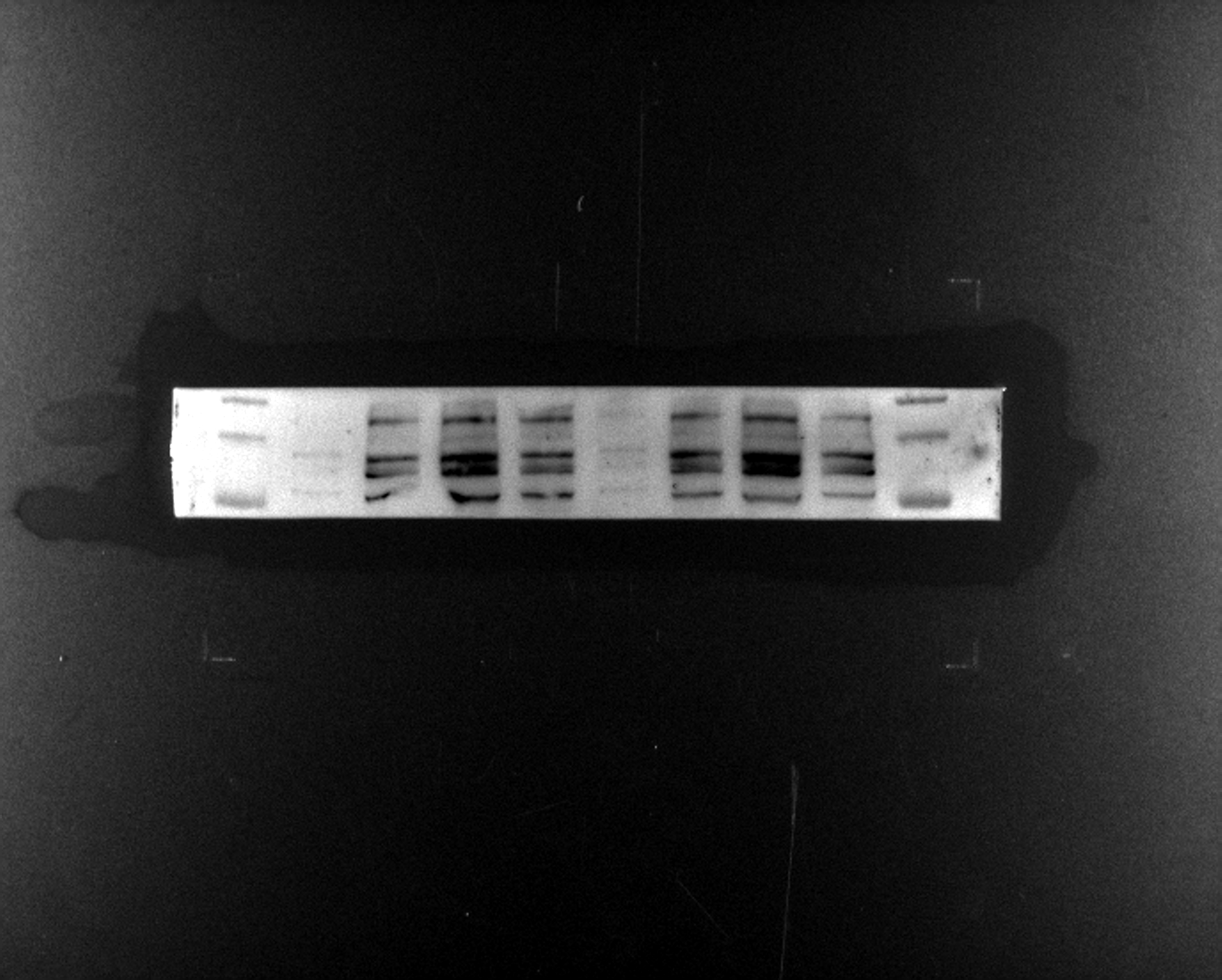

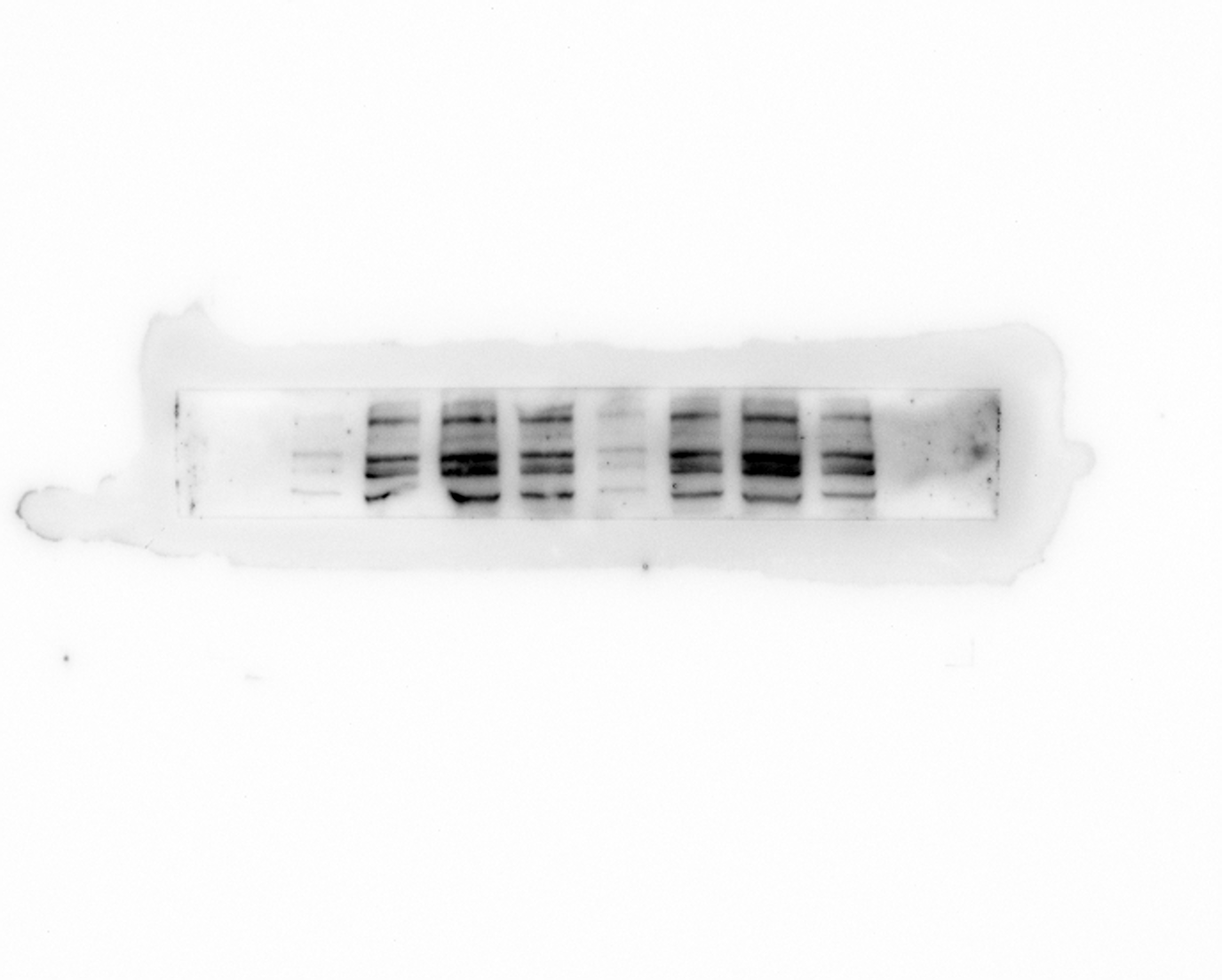


Antibody: MAML3

From right to left: MARKER, SGC7901 VECTOR, SGC7901 hsa_circ_0007967, BGC823 hsa_circ_0007967+mimic, BGC823 mimic, BGC823 VECTOR, BGC823 hsa_circ_0007967, BGC823 hsa_circ_0007967+mimic, BGC823 mimic, MARKER


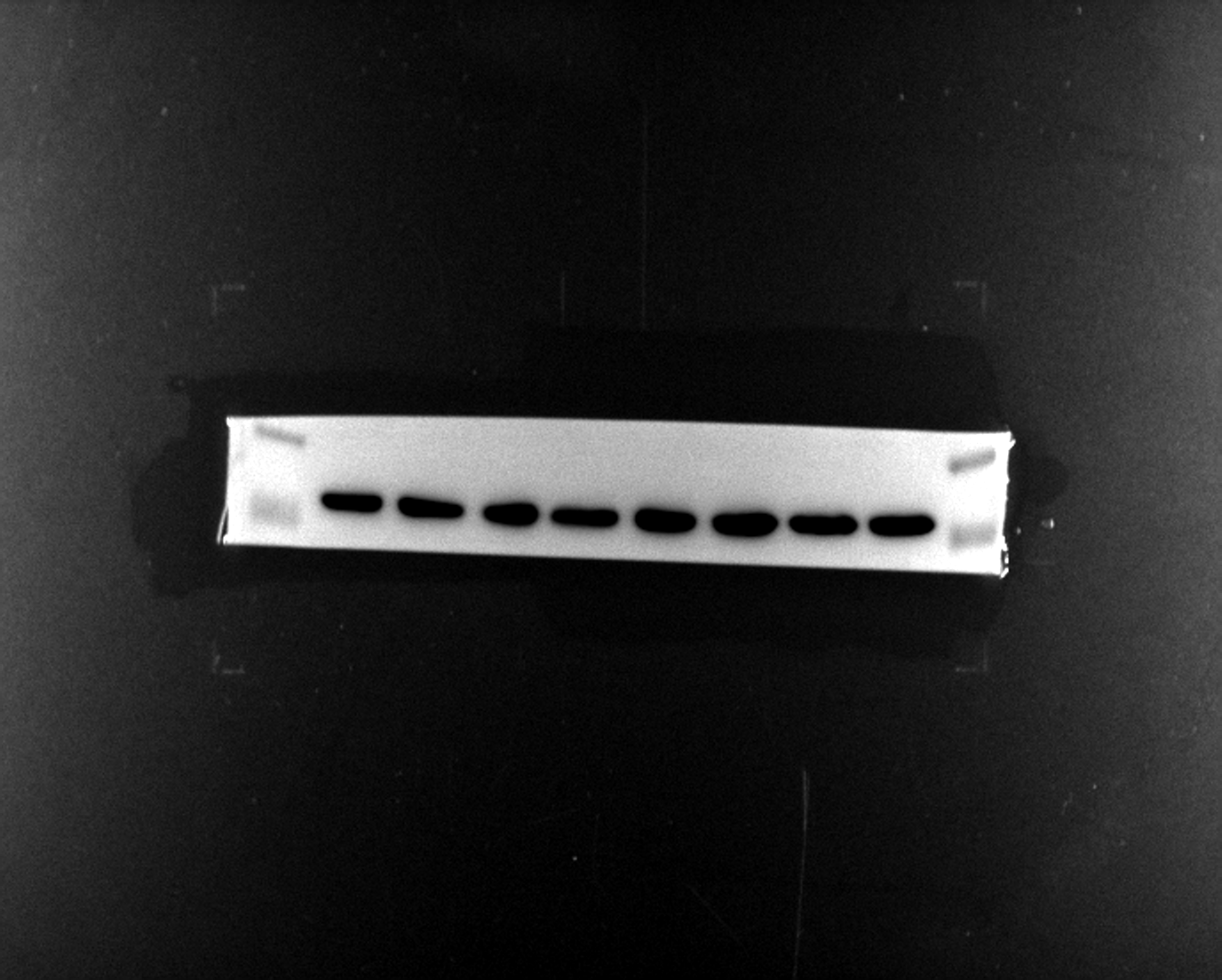

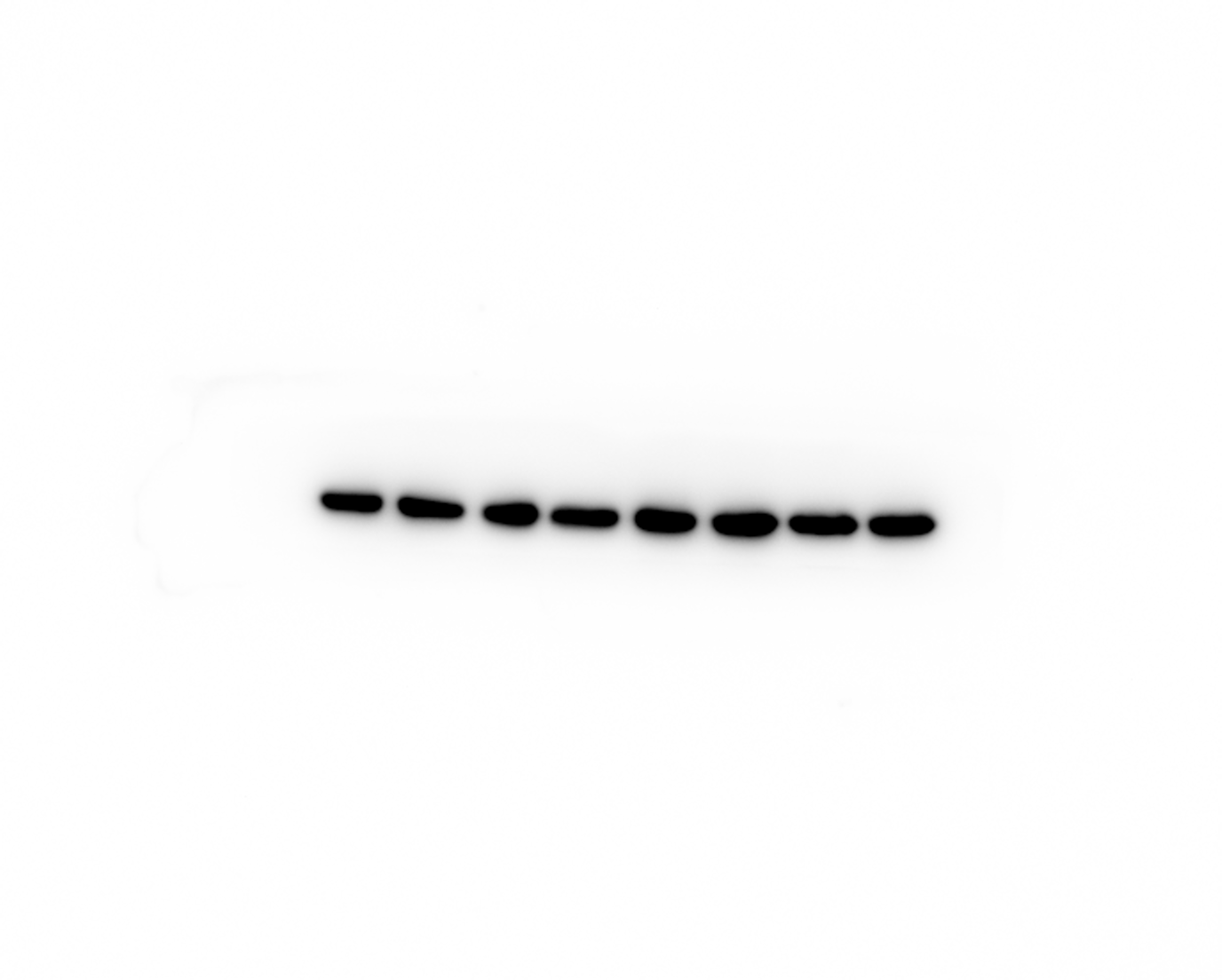


Antibody: GAPDH

From right to left: MARKER, SGC7901 VECTOR, SGC7901 hsa_circ_0007967, BGC823 hsa_circ_0007967+mimic, BGC823 mimic, BGC823 VECTOR, BGC823 hsa_circ_0007967, BGC823 hsa_circ_0007967+mimic, BGC823 mimic, MARKER
